# Characterization of a cold-active, detergent-stable metallopeptidase purified from *Bacillus sp*. S1DI 10 using Response Surface Methodology

**DOI:** 10.1371/journal.pone.0216990

**Published:** 2019-05-23

**Authors:** Drishtant Singh, Sharad Thakur, Seema Madhumal Thayil, Anup Kumar Kesavan

**Affiliations:** Molecular Microbiology Lab, Department of Molecular Biology and Biochemistry, Guru Nanak Dev University, Amritsar, Punjab, India; Universidade Nova de Lisboa Instituto de Tecnologia Quimica e Biologica, PORTUGAL

## Abstract

The colder regions of Earth are inhabited by cold-adapted microorganisms designated as psychrophiles that are known to produce cold-active enzymes, such as peptidases, chaperones, lipases, cellulases, and phosphatases. These types of enzymes are a major part of the market of industrial enzymes. Bacteria isolated from water samples collected from the Chamba region in the Himalayas were screened for peptidase production using skim milk agar plates. Among the peptidase-producing bacteria isolated, 20% of the isolates exhibited fast growth and maximum zones of clearance, and thus, were used for further studies. The 16S rDNA sequence analysis of isolate S1DI 10 identified it as a *Bacillus sp*. The peptidase was cloned in pET28a vector and expressed in *Escherichia coli* BL21(DE3) and the His-tagged recombinant protein was purified using Ni-NTA column. The purified peptidase of SIDI 10 was found to be an alkaline, cold-active peptidase with optimal enzyme activity at 10°C and pH 8. An approach of one variable at a time was used to further study the effect of various metal ions, organic solvents and detergents on the peptidase enzyme. The peptidase activity was enhanced in the presence of Fe^2+^ and Mn^2+^ (metal ions), hexane (organic solvent), SDS- sodium dodecyl sulfate (anionic detergent) and Tween 80 (nonionic detergent). Response surface methodology (RSM) was used to determine the cumulative effect of these five variables. A 2^5^ full factorial central composite design was applied for the five independent variables to determine the optimal combinations of these constituents at the maximum peptidase activity.

## Introduction

Microorganisms exist at temperatures ranging from -20°C to 120°C in extreme environments [1,2]. Microorganisms, which are adapted to extreme cold conditions, are called psychrophiles [3,4]. A diverse range of psychrophilic microorganisms, such as Gram-negative and Gram-positive bacteria, archaea, fungi and yeasts have been isolated from marine environments. These microorganisms and their metabolites have been found to have numerous industrial applications [5,6]. Cold-active peptidase has been isolated from different microorganisms inhabiting the Antarctic region, sub-Antarctic sediments, mountains, deep sea, glacier and the alpine environments [7,8].

Peptidases belong to one of the most important group of enzymes that have a major share in the industrial enzymes markets [5,9]. Peptidases are groups of hydrolases that hydrolyze peptide bonds (group 3 and subgroup 4 in the Enzyme Commission classification). Peptidases are classified into two types depending on their ability to cleave peptide bonds, i.e., exopeptidases that hydrolyze N- or C-terminal peptide linkages and endopeptidases that hydrolyze internal peptide linkages. On the basis of the functional groups present in their active sites, peptidases can be divided into different types viz. aspartic peptidases, cysteine peptidases, serine peptidases, threonine peptidases and metallopeptidases. Exopeptidases are further subdivided based on different characteristics, such as the cleavage of N- or C-terminal peptide linkages, the presence of charged moieties adjacent to the susceptible bond, optimal pH, substrate specificity, active site, sensitivity to inhibitors or their homology to peptidases previously characterized [10,11]. Most of the peptidases in the market are produced by *Bacillus sp*. *Bacillus* is an ubiquitous Gram-positive, rod shaped bacterium found in various environments [12–18]. This study was aimed at isolating a cold-active peptidase-producing bacterium from the waters of cold regions in the Himalayas to purify and characterize the peptidase and study the effect of various metal ions that may enhance the activity of the enzyme. Response Surface Methodology (RSM) was used to determine the optimal concentration of independent variables to obtain maximum activity of the peptidase.

## Material and methods

### Sample collection

Water samples were collected using aseptic conditions from the cold springs of Chamba, a hilly region in the Himalayas, in the Northern state of Himachal Pradesh, India. Samples were immediately transported to the laboratory and stored at 4°C until further processing. No specific permission was required for collection of water sample from the cold spring at Chamba, Himachal Pradesh, India. This location comes under rural area and doesn’t belong to any private or government organisation. Neither it comes under protected area nor any National park. The water body is not restricted for any use. Moreover, the organism isolated (*Bacillus sp*. S1DI 10) from the water sample is not endangered or a protected species.

### Isolation and screening of peptidase-producing bacteria

The isolation process involved two methods, including the direct processing and the concentration method. In direct processing, a known volume of samples was inoculated directly onto Luria Bertani (LB) agar plates, while in the concentration method, the same water samples were centrifuged, and the sediment/suspension at the bottom was inoculated onto the LB agar plates, which were incubated overnight at 20°C. Individual colonies from these plates were further subcultured onto modified skim milk agar plates, which contained 2% skim milk in LB agar media. These plates were maintained in a BOD incubator at 20°C for the next 3 days. The appearance of a clear zone around individual colonies on the plate indicated the production of peptidase. The individual colonies with a zone of clearance ~10 mm diameter were further inoculated on nutrient broth for bulk preparation, and -80°C stocks were prepared by mixing equal amounts of overnight cultures and sterile 50% glycerol for further studies.

### Morphological and biochemical characterization

For the morphological characterization using scanning electron microscopy (SEM), single colonies were inoculated in LB broth and incubated at 20°C overnight. The cells were harvested, washed three times with sterile distilled water and fixed in 2.5% glutaraldehyde at 4°C overnight. The cell pellets were resuspended after washing in sterile distilled water. One drop of suspension was transferred to a microscopic slide and air-dried. The smear was dehydrated serially with an alcohol gradient from 50%–100%. A thin layer of platinum was coated on the sample, and the samples were examined using SEM to study the morphology of the S1DI 10 isolate. The samples were further subjected to biochemical and physiological characterization following Bergey’s Manual of Systematic Bacteriology.

### Genomic DNA isolation

The isolate S1DI 10 was grown overnight, and the cells were harvested with Tris buffer (1 M Tris-HCl, 0.1 M EDTA, at 0.15 M NaCl, pH 8.0) at 2655 g at 37°C for 10 min. The DNA was isolated as described by Valicente et. al. with slight modifications [19]. The pellet resuspended in Tris buffer was treated with 16 μL of 100 mg/mL lysozyme and incubated at 37°C for 30 min. One microliter of 10 mg/mL RNase was added to the solution and incubated for 15 min at 37°C. The suspension was treated with SDS at 65°C for 20 min and by Proteinase K at 65°C overnight. A total of 1.36 M NaCl was added, and the supernatant was collected after centrifugation at 19357 g for 10 min at 37°C. An equal volume of alcohol was added to the supernatant to precipitate the DNA, and the precipitate was washed in 70% alcohol and resuspended in TE buffer.

### 16S rRNA gene characterization

The 16S rRNA gene was amplified in a 20 μL reaction mixture containing Taq buffer (10X), 10 μM each of forward and reverse primer, 10 mM dNTPs, 20 ng/μL DNA, and 5U Taq Polymerase (Genei, India). The previously described 16Sr DNA primers PF1 5’-CAGGCCTAACACATGCAAGTC-3’ and PR1 5’-GGGCGGWGTGTACAAGGC-3’ were used [20]. The PCR reaction was performed in a Thermocycler (Agilent Technologies, USA) with an initial denaturation step of 94°C for 3 min, followed by 35 cycles of denaturation at 94°C for 30 seconds, annealing at 59°C for 30 seconds and extension at 72°C for 45 seconds. A final step of extension was performed at 72°C for 10 min. The PCR-amplified product was purified using a PCR/Gel Purification Kit (IBI, Scientific, USA) following the manufacturer’s instructions and sequenced (First Base Laboratories, Malaysia). The 16S rDNA sequence obtained in this study was subjected to a BLAST search in the NCBI database to identify similar sequences. The sequences of the nearest relatives were obtained, and a phylogenetic tree was constructed using Mega 7.0.14 software to chart the evolutionary lineage. The 16S rDNA sequence was submitted to the NCBI Gene Bank, BankIt (www.ncbi.nlm.nih.gov/Banklt/).

### Production and purification of the peptidase

To produce peptidase, the isolates were grown overnight in LB media at 20°C, and 5% inoculum was further subcultured in media containing glucose (0.5%), casein (0.5%), peptone (0.5%), MgSO_4_.7H_2_O (0.75%), KH_2_PO_4_ (0.5%) and FeSO_4_.7H_2_O (0.01%), pH 7.0. The cultures were incubated at 20°C at 180 rpm in an orbital shaker and harvested at different time intervals between 12 hours to 72 hours post inoculation. The harvested cultures were centrifuged at 10621 g for 15 min at 4°C to obtain a clear supernatant. The supernatant was passed through 0.22 μm PES membrane filters for further purification. A time course experiment was devised to study the peptidase enzyme production by the crude extract. The cell-free supernatant was precipitated with ammonium sulfate and pelleted by centrifugation at 10621 g for 10 min at 4°C. The pellet was dissolved in 50 mM phosphate buffer (pH 8.0) and dialyzed against the same buffer overnight at 4°C. The dialysed sample was further purified by gel filtration chromatography using a Sephadex-G100 column (Sigma, USA) pre-equilibrated with 50 mM phosphate buffer (pH 8.0) and eluted at a flow rate of 0.25 mL/minute. The collected fractions were further subjected to SDS-PAGE and native PAGE to determine the molecular weight of the enzyme and were assayed for peptidase activity as previously described [21]. The fractions with maximum purity and the highest activity were pooled and stored for further studies.

### Cloning, expression and purification of cold peptidase

The primers for peptidase gene were designed on the basis of conserved nucleotide sequences; **PDFP1** 5'**-**ATGAAAAAAGT**R**TCAATTCGG**K**CTGTATT**R**-3' and **PDRP1** 5'-TTA**Y**T**K**AATTTC**R**AATTCTCCTAAAGAACC**D**GT-3'. The PCR amplification of the gene was done using Q5 High Fidelity DNA Polymerase in a Thermocycler (Agilent Technologies, USA) with an initial denaturation step of 98°C for 30 seconds, followed by 35 cycles of denaturation at 98°C for 10 seconds, annealing at 64°C for 30 seconds and extension at 72°C for 2 min & 10 seconds. A final step of extension was performed at 72°C for 10 min. The PCR-amplified product was purified using a PCR/Gel Purification Kit (IBI Scientific, USA) following the manufacturer’s instructions and sequenced (First Base Laboratories, Malaysia). The amplified product was sequenced using the same primers described above. The forward and reverse sequences were aligned using CodonCode Aligner 4.2.5 to obtain the whole length nucleotide sequence of the cold peptidase gene. After obtaining the sequence of the gene, NCBI database search was carried out for identification and similarity check with other extracellular peptidases. A nested PCR using primers with restriction enzyme site added to the 5' end (**PDFP Sal1** 5'—ACG CGT CGA CGT ATG AAA AAA GTA TCA ATT CGG—3' and **PDRP XhoI** 5—CCG CTC GAG TTA TTT AAT TTC GAA TTC TCC—3') were designed to amplify the region spanning open reading frame for cold peptidase. PCR amplification of the peptidase gene was done in a Thermocycler (Agilent Technologies, USA) with the procedure described above at 62°C annealing temperature. The PCR-amplified product was purified and digested with restriction enzymes; SalI and XhoI (NEB, USA) at 37°C for 4 hours. pET 28a plasmid (mention the company) was digested overnight with the aforementioned restriction enzymes at 37°C. The digested PCR product and vector were ligated using T4 DNA Ligase (NEB, USA) at 16°C for 16 hours and transformed into *DH5α* strain of *Escherichia coli*. The transformed cells were plated on Kanamycin (50 μg/ml) supplemented LB Agar plates.After incubation at 37C for 18 hours, clones with successful recombination showing Kanamycin resistance were selected and multiplied. Plasmid DNA isolated from the selected clone was transformed into BL21(DE3) strain of *E*.*coli*. The transformed BL21(DE3) cells were grown overnight at 37°C and subcultured in fresh medium.1mM IPTG (Isopropyl β -D-1-thiogalactopyranoside) containing media was used for overexpression of peptidase. The cells were lysed and the peptidase was purified by affinity chromatography using Ni-NTA beads (QIAGEN, Germany) as per the manufacturer’s instructions.

### Quantitative assay for the estimation of peptidase

The peptidase activity of the culture supernatant was measured by the hydrolysis of casein as described previously [21]. Briefly, known quantities of the crude extracts were added to 1% casein (in 50 mM phosphate buffer, pH 7.0), and the mixture was incubated for 20 min at varying temperatures. The reaction was terminated by the addition of 1 mL of 10% TCA and incubated at room temperature for 15 min followed by centrifugation at 10621 g for 10 min. The supernatant was collected and buffered with 2.5 mL of 400 mM Na_2_CO_3_. One mL of FC reagent (diluted 3 times) was immediately added, and the mixture was incubated in the dark at room temperature for 30 min. The absorbance was measured at 660 nm to predict enzymatic activity using a blank and a tyrosine standard solution as reaction controls [22].

One unit of the enzyme is defined by the amount of peptidase that produces 1 μmole/mL of tyrosine equivalents per minute under the standard conditions.

$$\text{Units}/\text{mL}\;\text{enzyme}=\frac{\text{X}\;\mu \text{mol}\;\text{of}\;\text{tyrosine}\;\text{equivalents}\;\text{released}\;\text{x}\;\text{total}\;\text{volume}\;\text{of}\;\text{assay}}{\text{Vol}.\;\text{of}\;\text{enzyme}\;\text{used}\;\text{x}\;\text{length}\;\text{of}\;\text{assay}\;\text{x}\;\text{vol}.\;\text{used}\;\text{in}\;\text{the}\;\text{colorimetric}\;\text{determination}}$$

### Effect of temperature on the peptidase activity

To study the effect of temperature on the activity of the peptidase, the substrate (casein) and purified enzyme were preincubated separately at predetermined temperatures, and the mixture of the substrate and enzyme was then incubated at 5°C, 10°C, 15°C and 20°C. The peptidase activity was determined using the equation described above.

### Effect of pH on the peptidase activity

After identifying the optimal temperature for peptidase activity using the crude enzyme, studies were designed to determine the optimal pH for enzyme activity. For this assay, the enzyme activity was recorded at pH 6, 7, 8, 9, 10 and 11. Known quantities of substrate were resuspended in different buffers to determine the activity at a particular pH. Fifty millimolar phosphate buffer was used for studies at pH 6 and pH 7. Fifty millimolar Tris-HCl buffer was used for studies at pH 8 and pH 9, and 50 mM sodium bicarbonate buffer was used for the studies at pH 10 and pH 11. The enzyme was mixed with an equal volume of substrate at different pH values, and the mixture was incubated at 10°C to measure the peptidase activity.

### Effect of inorganic and organic moieties on the peptidase activity

#### Metal ions

The effect of the metal ions Fe^2+^, Mn^2+^, Zn^2+^, Co^2+^, Ni^2+^, Mg^2+^, Ca^2+^, Cu^2+^, Na^+^ and K^+^ on enzyme activity was recorded using varying concentrations of metal ions (2 mM, 5 mM, 10 mM, 20 mM and 50 mM). The mixture was incubated prior to the addition of the enzyme at 10°C for 30 min. After preincubation, the substrate was added to the enzyme-metal ion mixture and assayed for peptidase activity at optimal temperature and pH. The activity of purified enzyme without any metal ions was used as the control, and the residual activity was calculated.

#### Organic solvents

The effect of organic solvents on enzyme activity was examined using varying concentrations of the organic solvents ethanol, methanol, isopropanol, hexane, acetone, butanol, toluene, chloroform, DMSO (dimethyl sulfoxide) and PEG (polyethylene glycol). The enzyme-solvent mixture was preincubated at 10°C for 30 min followed by the peptidase assay. The specific activity of the enzyme without solvents was used as the control. A negative control was established using organic solvents and substrate in the absence of peptidase to identify the activity of the solvent, if any, on the substrate.

#### Detergents

The effect of detergents on enzyme activity was assayed using known concentrations of anionic, cationic, nonionic and chaotropic detergents, including urea, Tween 80, Triton X-100, SDS (sodium dodecyl sulfate) and CTAB (cetrimonium bromide). The enzyme and detergent mixture was preincubated at 10°C for 30 min and examined for peptidase activity as described above. The specific activity of the purified enzyme at the optimal temperature and pH without any detergent was used as the control.

#### Known peptidase inhibitors

The effect of known peptidase inhibitors (PI) on enzyme activity was assayed using varying concentrations of PI as per the standardized protocol. The inhibitors used in this study were EDTA (ethylenediaminetetraacetic acid), PMSF (phenylmethylsulfonyl fluoride), leupeptin, pepstatin and iodoacetamide. The experiments were repeated three times, and a statistical analysis of the experimental data was conducted with a Tukey’s test using MINITAB 14.12.0.

#### Enzyme kinetics

The kinetic parameters Km and V_max_ were determined using different concentrations (0.2 mM– 20 mM) of substrate (casein) using a Lineweaver-Burk plot (GraphPad Prism 7).

### Design of the experiment for enhanced enzymatic activity

A one variable at a time approach was utilized to determine the effect of various metal ions, organic solvents and detergents on the peptidase enzyme activity. Based on their ability to enhance the enzyme activity, five factors, including Mn^2+^, Fe^2+^, hexane, Tween-80 and SDS were identified.

RSM (Response Surface Methodology) was employed as the next step to study the combined effect of two independent variables on enzyme activity, while keeping the other three variables at a zero level. 3D response surface plots were prepared using Tera Plot 1.4.03 (Kylebank Software Ltd.) to determine the relation between the two variables. A 2^5^ full factorial central composite design for five independent variables was employed to calculate the optimal concentrations in different combinations of the variables described above for maximum enzyme activity. The experiments were designed using Design Expert 7.0.0 (Stat-Ease) for 5 independent variables at 5 levels. The range and levels of the five independent variables is shown in S1 Table. A total of 50 experiments were performed employing five variables each at five levels. The second order polynomial model was expressed as the regression function in the following equation:

$$\begin{array}{l} \text{Y}=\beta_{0}+\beta_{1}{\text{X}}_{1}+\beta_{2}{\text{X}}_{2}+\beta_{3}{\text{X}}_{3}+\beta_{4}{\text{X}}_{4}+\beta_{5}{\text{X}}_{5}+\beta_{12}{\text{X}}_{1}{\text{X}}_{2}+\beta_{13}{\text{X}}_{1}{\text{X}}_{3}+\beta_{14}{\text{X}}_{1}{\text{X}}_{4}+\beta_{15}{\text{X}}_{1}{\text{X}}_{5}+\beta_{23}{\text{X}}_{2}{\text{X}}_{3}+\beta_{24}{\text{X}}_{2}{\text{X}}_{4}+\\ \beta_{25}{\text{X}}_{2}{\text{X}}_{5}+\beta_{34}{\text{X}}_{3}{\text{X}}_{4}+\beta_{35}{\text{X}}_{3}{\text{X}}_{5}+\beta_{45}{\text{X}}_{4}{\text{X}}_{5}+\beta_{11}{\text{X}}_{11}^{2}+\beta_{22}{\text{X}}_{22}^{2}+\beta_{33}{\text{X}}_{33}^{2}+\beta_{44}{\text{X}}_{44}^{2}+\beta_{55}{\text{X}}_{55}^{2} \end{array}$$

where Y is the peptidase activity (response) in units/mL; β_0_ is the intercept value; β_1_, β2, β_3_, β_4_ and β_5_ are the linear coefficients; β_12_, β_13_, β_14_, β_15_, β_23_, β_24_, β_25_, β_34_, β_35_ and β_45_ are the interaction coefficients; β_11_, β_22_, β_33_, β_44_ and β_55_ are the quadratic coefficients, and X_1_, X_2_, X_3_, X_4_ and X_5_ are independent variables. The experimental data and the model were analyzed statistically using an ANOVA (analysis of variance). The statistical model and its predictions were validated by conducting the suggested experiments in triplicate at optimal temperature and pH.

## Results

### Isolation and screening of the peptidase-producing bacteria

The bacterial strains with a clear zone of 10 mm diameter and above on skim milk agar plates (S1 Fig) were selected for further growth and kinetic studies to isolate fast growers.

### Morphological and biochemical characterization

**Fig 1:**
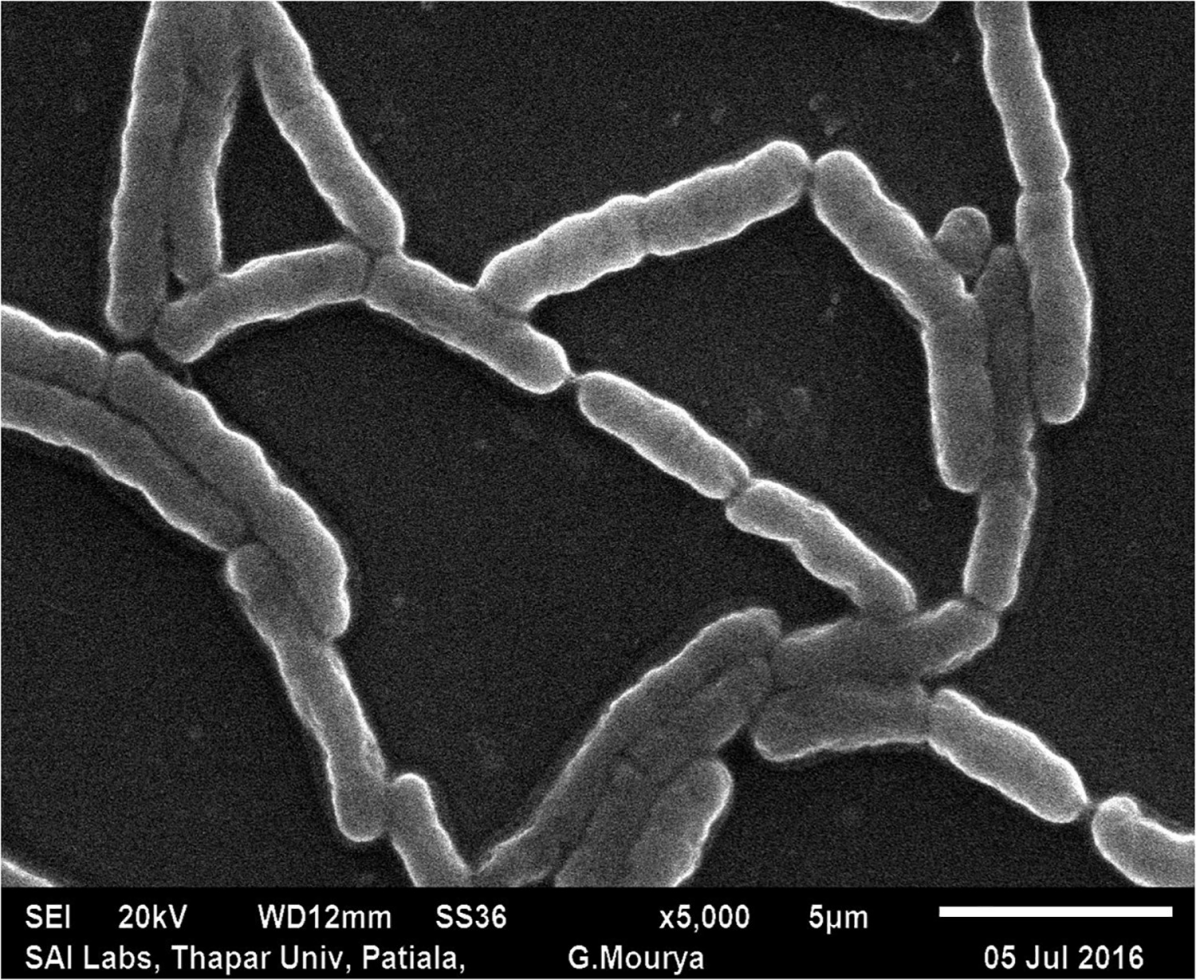
SEM of *Bacillus sp*. S1DI 10.

The morphology of the isolate using light microscopy revealed a Gram-positive, rod shaped bacterium. It was further analyzed for characteristic spore formation using SEM (Fig 1). S2 Table represents the various biochemical characteristics of S1DI 10.

### 16S rRNA gene characterization

**Fig 2:**
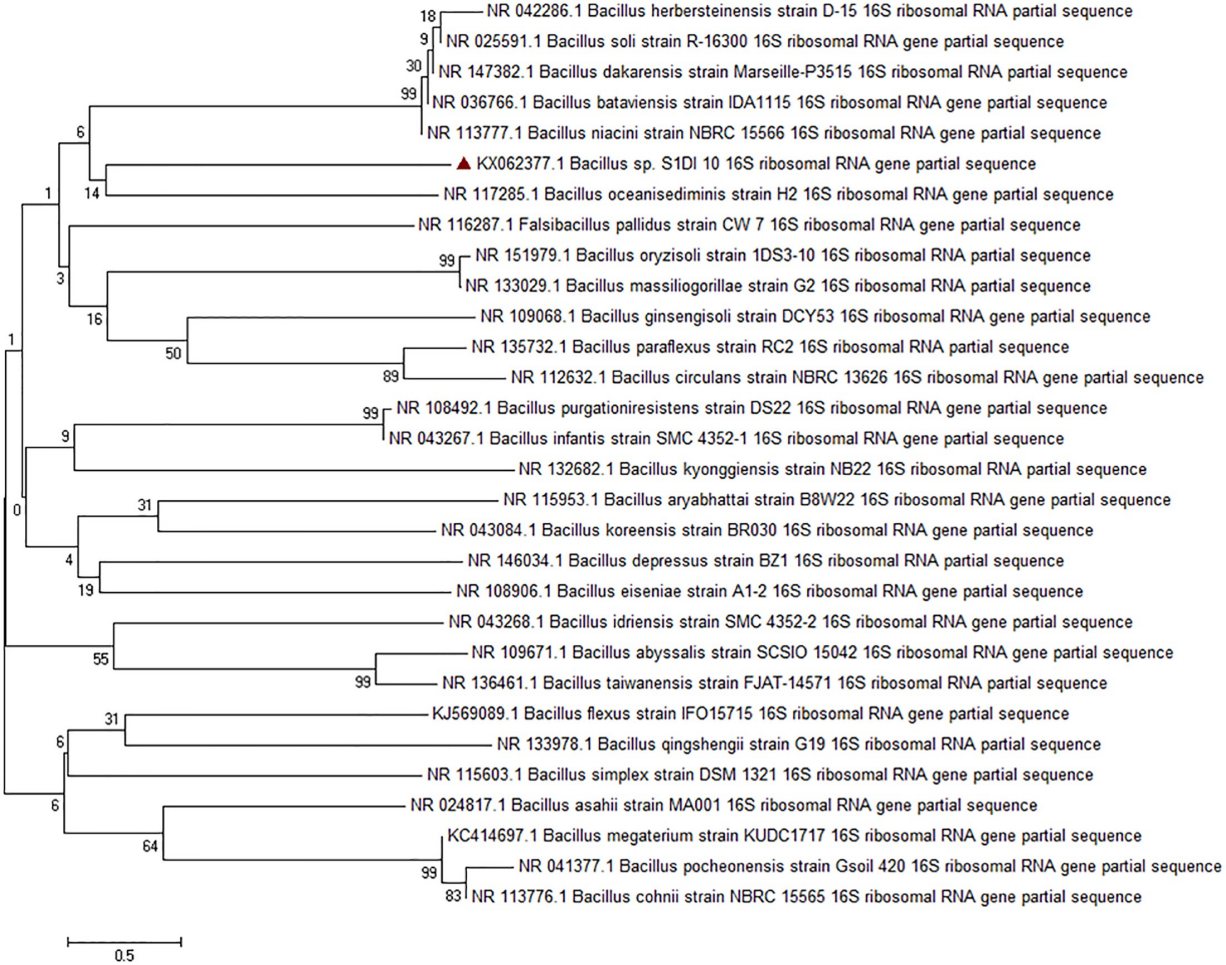
Phylogenetic tree of *Bacillus sp*. S1DI 10 based on 16sRNA gene sequence.

A 1450 bp amplicon was obtained by PCR amplification of the 16S rDNA from S1DI 10 (S2 Fig). The alignment of the 16S rDNA sequence against the NCBI database suggested that S1DI 10 is a *Bacillus sp*. (GenBank Accession number KX062377). The phylogenetic tree analysis with the closest relative elucidated that *Bacillus sp*. S1DI 10 clusters with *Bacillus oceanisediminis*. The Neighbor-Joining method was used to deduce the evolutionary history [23] in which 30 nucleotide sequences were used to construct the phylogenetic tree with the help of MEGA7 [24]. Fig 2 shows the optimal tree with the sum of the branch length = 33.81277813. The associated taxa clustered together in the bootstrap test (1000 replicates) represent the percentage of replicate trees shown next to the branches [25]. The dendrogram is drawn to scale with branch lengths in the same units as those of the evolutionary distances used to deduce the phylogenetic tree. The maximum composite likelihood method was used to enumerate the evolutionary distance [26].

### Production and purification of the peptidase

The specific activity of the crude enzyme fractions collected at different time points was found to be 80 U, 115 U, 125 U, 132 U, 139 U and 141 U per mL of the supernatant at 12, 24, 36, 48, 60 and 72 hours, respectively (S3 Fig). The protein was purified using ammonium sulfate precipitation followed by dialysis. The dialyzed sample was further concentrated and purified using a Sephadex G-100 column (S4 Fig), which resulted in 5.2-fold purification and 17% recovery of the peptidase (S3 Table). The purified peptidase was analyzed using SDS-PAGE and native PAGE resulting in a protein band with a molecular weight of ~40 kDa (S5 Fig) and ~80 kDa (S6 Fig), respectively.

### Cloning, expression and purification of peptidase

**Fig 3:**
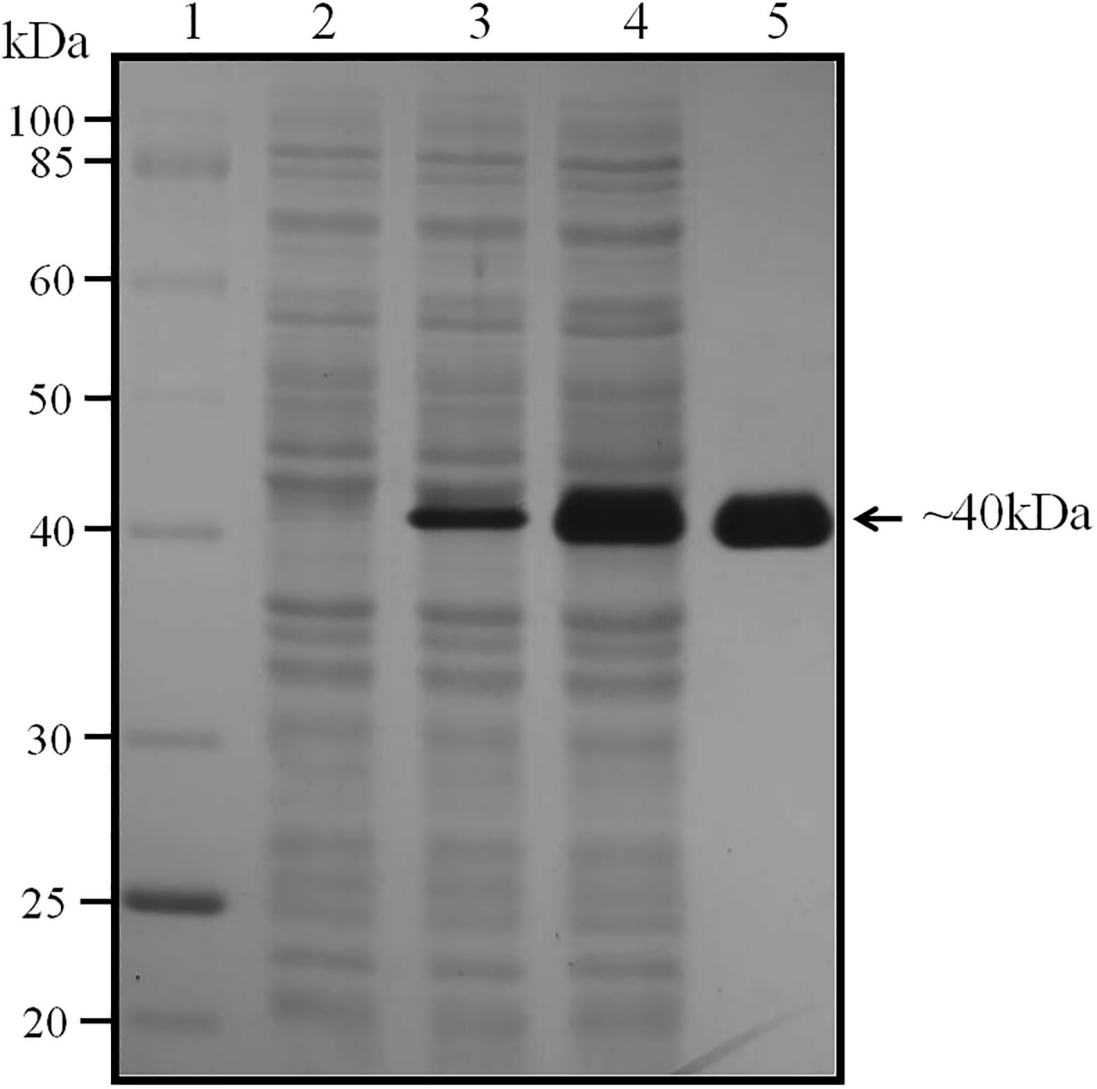
SDS PAGE analysis of purified peptidase expressed in BL21(DE3) strain of *E*. *coli*. (Lane 1—Protein Standard (NEB, USA), Lane 2—Negative Control (*E*. *coli BL21DE3*), Lane 3—Uninduced clone, Lane 4—Induced clone, Lane 5—Purified peptidase).

The amplification of cold peptidase gene from S1DI 10 resulted in a ~2400bp PCR product. S7(A) Fig depicts the PCR product of peptidase gene. S7(B) Fig depicts the digested products from the transformed pET 28a plasmid. The transformed *E*.*coli* DH5α colonies were patched on skim milk agar plates and the clones which gave zone of clearance above 10mm were selected for sequencing. The forward and reverse sequences were aligned using CodonCode Aligner 4.2.5 to obtain the whole length nucleotide sequence of the cold peptidase gene (GenBank Accession no. MK783941). The gene sequence of our cold peptidase showed 100% similarity to minor extracellular protease from *Bacillus megaterium QM B1551* and Peptidase S8 from *Bacillus sp*. *Y-01*. However, the phylogenetic tree analysis with the closest relative showed that cold peptidase clustered with protease from *Bacillus megaterium QM B1551* (S8 Fig). The cold peptidase was successfully expressed in *E*.*coli* BL21(DE3) and purified by affinity chromatography using Ni-NTA beads (Fig 3). This purified peptidase was used for further characterisation.

### Effect of temperature on the peptidase activity

**Fig 4:**
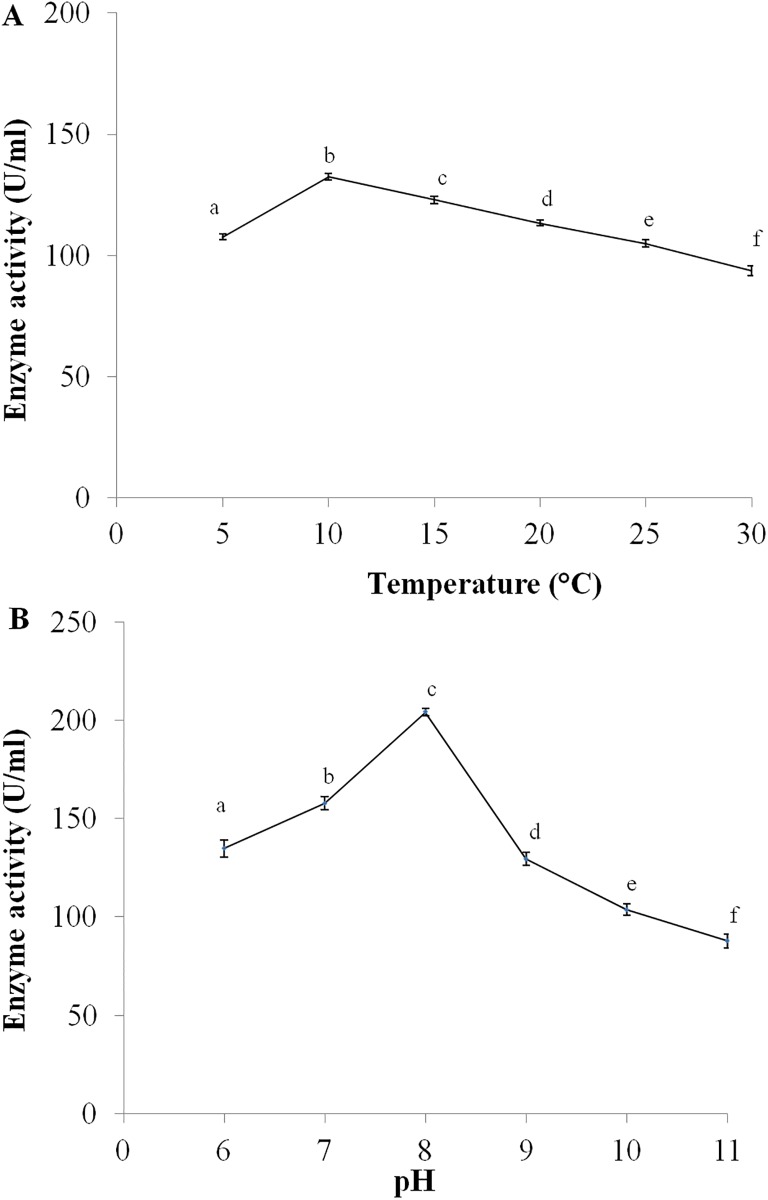
Effect of (A) Temperature (HSD = 4.78, F = 242.04*, p≤0.5) and (B) pH (HSD = 10, F = 701.52*, p≤0.5) on the activity of peptidase isolated from *Bacillus sp*. S1DI 10.

The enzyme showed activity over a broad temperature ranging from 5°C to 30°C. The enzyme is psychrophilic, since the optimal temperature for peptidase activity was found to be 10°C, and the enzyme exhibited significant activity near 0°C. The specific activity of the purified enzyme was found to be 132 U/mL at 10°C. Fig 4A shows the enzyme activity and the range of temperature used for the test.

### Effect of pH on the peptidase activity

The enzyme was found to be active from pH 6 to pH 11 (Fig 4B) with optimal activity at pH 8. The specific activity at the optimum pH and temperature was found to be 204 U/mL, and was considered to be 100% residual activity, while conducting further assays to analyze the effect of metal ions, organic solvents and detergents on enzyme activity.

### Effect of inorganic and organic moieties on the peptidase activity

#### Effect of metal ions on the peptidase activity

**Fig 5:**
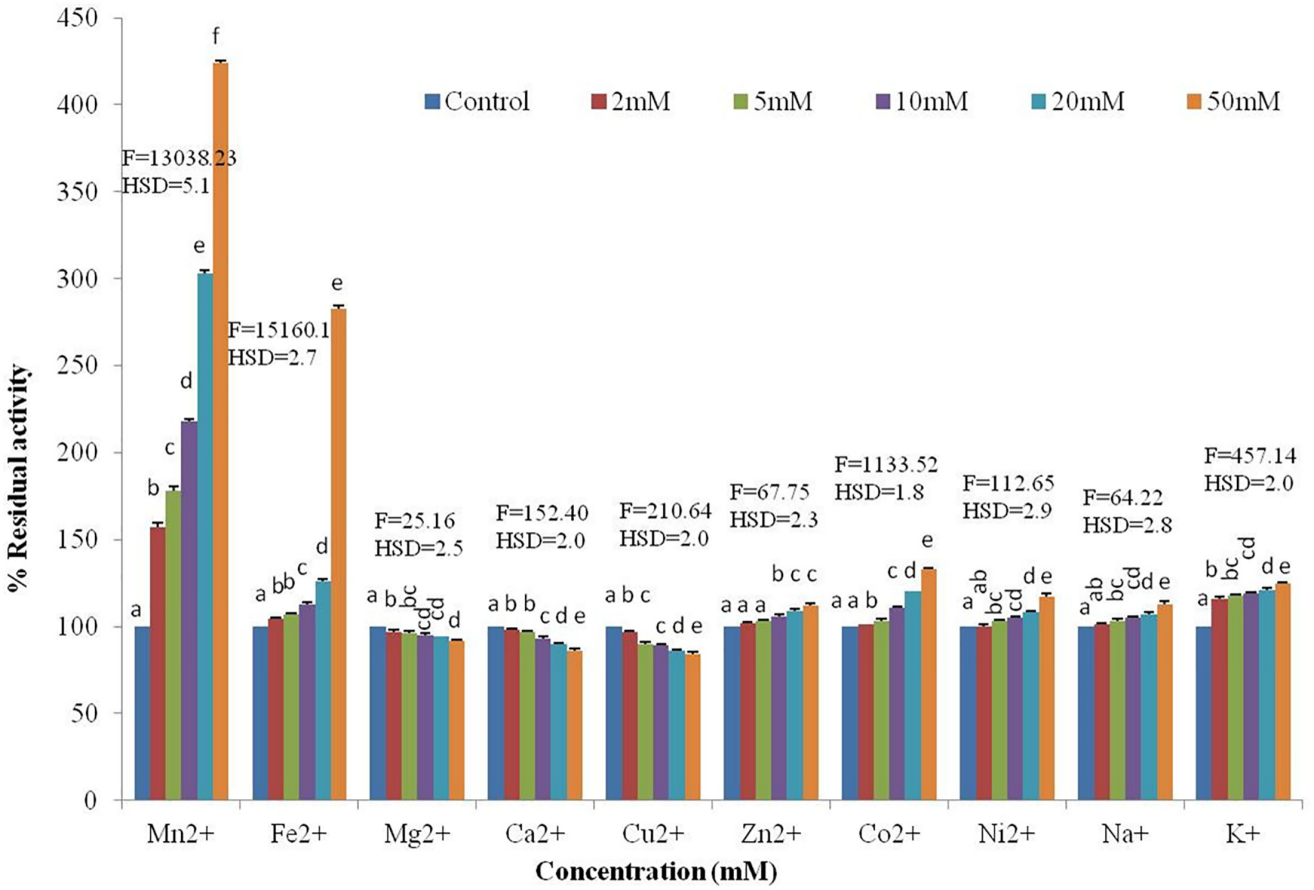
Effect of various metal ions on the activity of Peptidase produced by *Bacillus sp*. S1DI10 (F = 8929.03*, p≤0.05). The residual activity of control was taken as 100%. *Significant at 5% level of significance.

The enzyme activity was enhanced by Fe^2+^, Mn^2+^, Zn^2+^, Co^2+^ and Ni^2+^. However, the enzyme activity was slightly inhibited by Mg^2+^, Ca^2+^ and Cu^2+^ (Fig 5). The enzyme activity increased up to 280% with 50 mM Fe^2+^ and to 400% with 50 mM Mn^2+^. However, the enzyme activity decreased by 8%, 14% and 16% with 50 mM Mg^2+^, Ca^2+^ and Cu^2+^, respectively. In addition, there was a slight increase in activity with 50 mM concentrations of Zn^2+^(112%), Co^2+^(133%) and Ni^2+^(117%). In addition, a slight increase in activity with the monovalent ions 50 mM Na^+^(113%) and 125% by 50 mM K^+^(125%) was observed.

#### Effect of organic solvents on the peptidase activity

**Fig 6:**
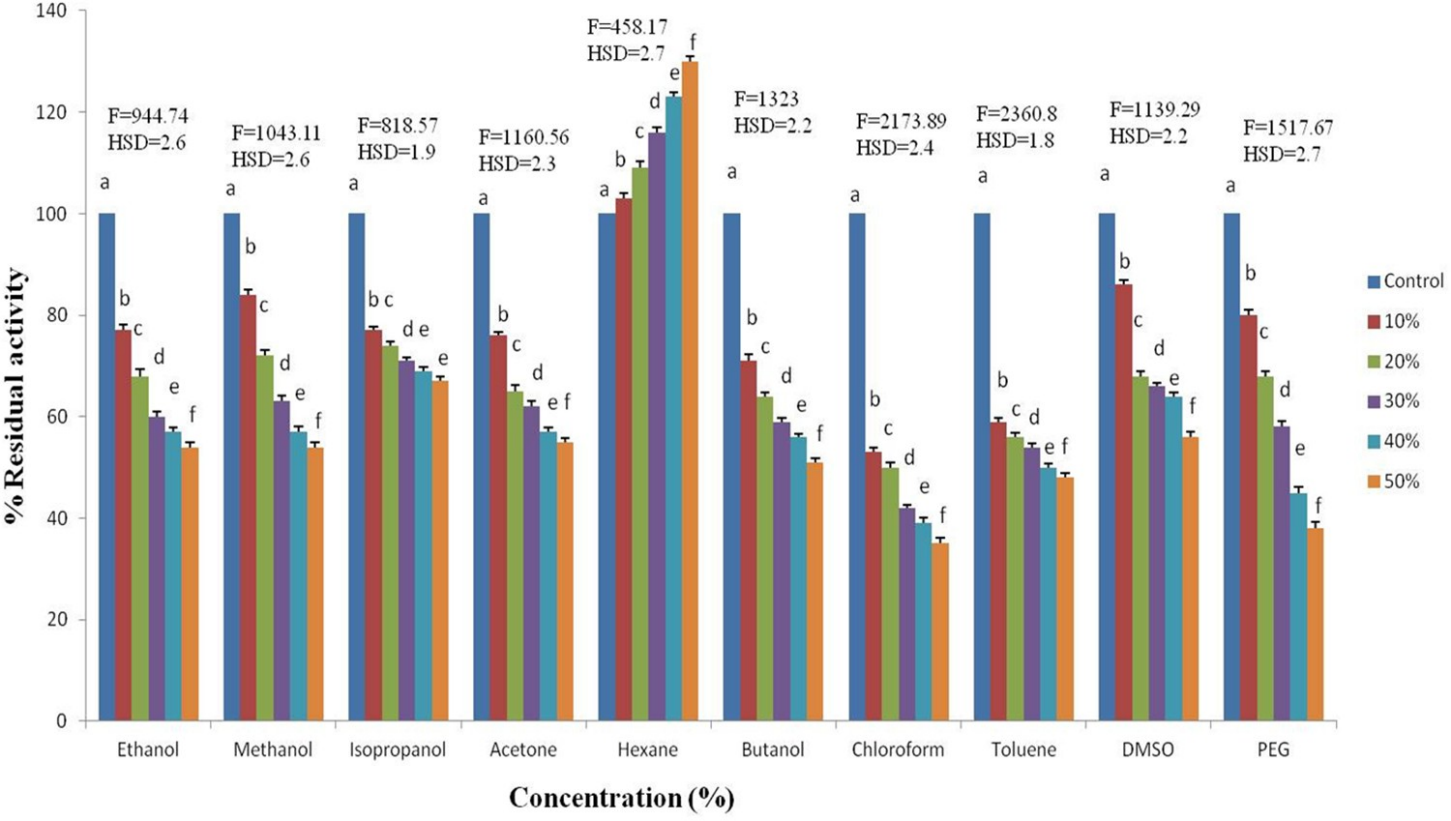
Effect of various organic solvents on the activity of peptidase produced by *Bacillus sp*. S1DI10 (F = 1394*, p≤0.05). *Significant at 5% level of significance.

The enzyme activity at the optimal pH and temperature was enhanced up to 130% in hexane (50%), while the other organic solvents ethanol, methanol, isopropanol, acetone, butanol, toluene, chloroform, DMSO (dimethyl sulfoxide) and PEG (polyethylene glycol) decreased the enzyme activity in 30 min (Fig 6). The enzyme activity was decreased by 33% in the presence of 50% isopropanol. Approximately 50% of the enzyme activity was lost in the presence of 50% of methanol, ethanol, acetone, butanol, toluene and DMSO. More than 60% of the activity of enzyme was lost in the presence of 50% chloroform and PEG.

#### Effect of detergents on the peptidase activity

**Fig 7:**
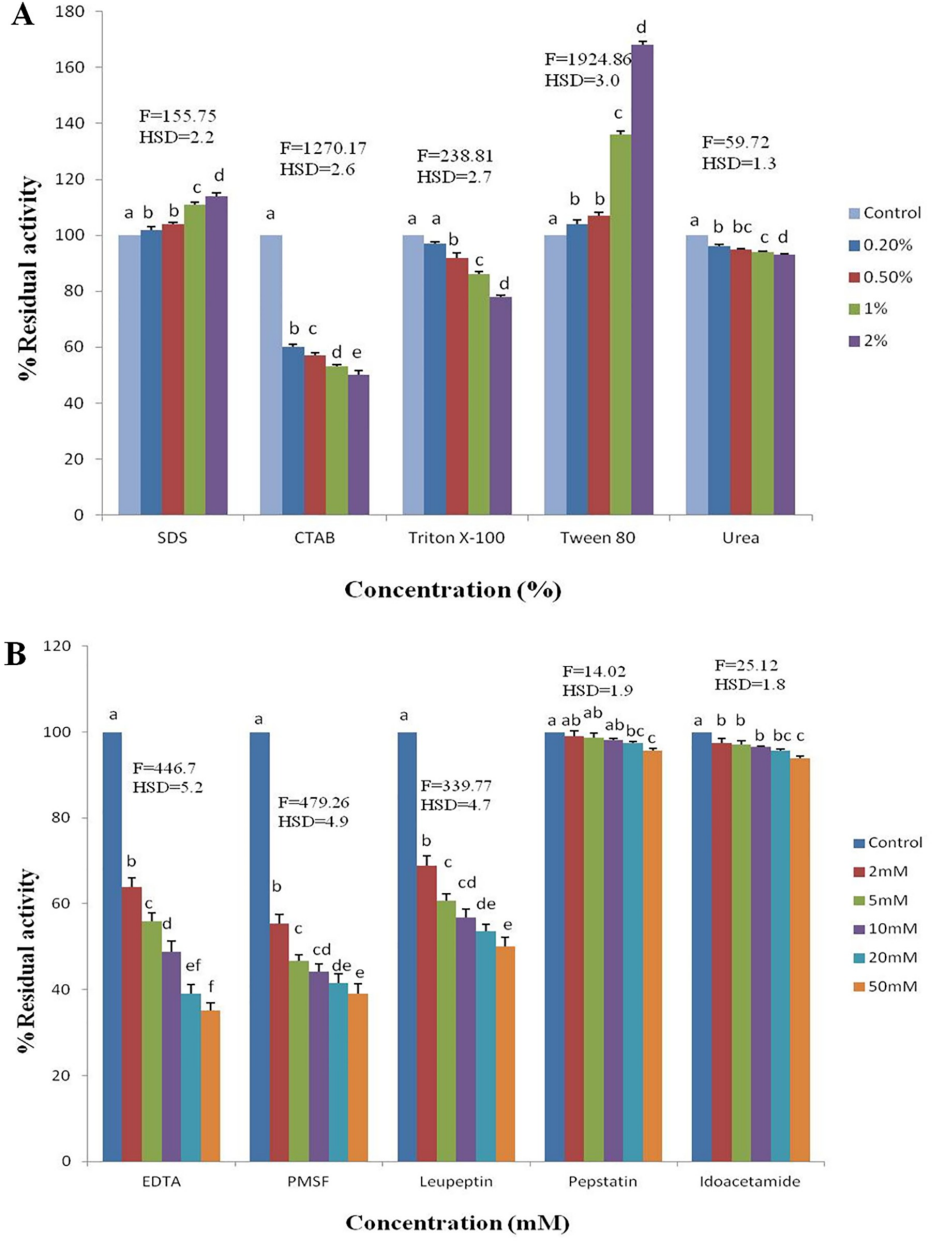
S1DI10 peptidase inhibition by varying concentrations of (A) Detergents (F = 2216.85*, p≤0.05) and (B) Known peptidase inhibitors (F = 676.84*, p≤0.05). *Significant at 5% level of significance.

The activity of the enzyme increased up to 168% in the presence of 2% Tween 80. The enzyme activity was also enhanced up to 114% with 2% SDS (sodium dodecyl sulfate). The enzyme activity decreased by 50% in 2% CTAB (cetrimonium bromide) and by 22% in 2% Triton X-100 after incubation for 30 min. The enzyme was relatively stable in 2% urea and retained 93% of its activity (Fig 7A).

#### Effect of inhibitors on the peptidase activity

The activity of the enzyme decreased by 65% in the presence of 50 mM EDTA. In addition, the enzyme activity was found to decline to 60% in the presence of 50 mM PMSF and to 50% in the presence of 50 mM leupeptin. Alternatively, pepstatin and iodoacetamide had no significant effect on the enzyme activity. Approximately 95% and 93% of the enzyme activity was retained in the presence of 50 mM pepstatin and iodoacetamide, respectively (Fig 7B). A significant finding in this assay was the result that following pre-incubation with EDTA, the enzyme activity was retained in the presence of Mn^2+^ without any significant decline.

### Enzyme kinetics

Michaelis-Menten kinetics were observed for the proteolysis of casein by the cold protease. The Km and V_max_ values obtained from Lineweaver-Burk plot (S9 Fig) were 2.16 mM and 0.013 mM/ml/min, respectively.

### Optimization of the peptidase activity using Response Surface Methodology

The cumulative effect of five independent variables, including Mn^2+^, Fe^2+^, hexane, Tween-80 and SDS, on peptidase activity was studied using CCD via RSM. The Central Composite Design matrix and the observed and predicted values of enzyme activity are listed in S4 Table. The model F-value of 6.69 obtained by ANOVA suggested that the model was significant (Table 1). The coefficients obtained by regression analysis and their significance are shown in S5 Table. The experimental results were consistent with the model constructed as evident from the “Lack-of-Fit F-value” of 2.7. The final equation for peptidase activity (Y) in terms of the coded factors was obtained as follows:

$$\begin{array}{l} \text{Y}=790.81+\;116.61\;{\text{X}}_{1}+\text{52}.87\;{\text{X}}_{2}+59.28\;{\text{X}}_{3}+71.65\;{\text{X}}_{4}+77.85\;{\text{X}}_{\text{5}}+10.88\;{\text{X}}_{1}{\text{X}}_{2}+8.43\;{\text{X}}_{1}{\text{X}}_{3}+19.54\;{\text{X}}_{1}{\text{X}}_{4}\\ +13.45\;{\text{X}}_{1}{\text{X}}_{5}+1.83\;{\text{X}}_{2}{\text{X}}_{3}+3.92\;{\text{X}}_{2}{\text{X}}_{4}+13.29\;{\text{X}}_{2}{\text{X}}_{5}+8.62\;{\text{X}}_{3}{\text{X}}_{4}+6.98\;{\text{X}}_{3}{\text{X}}_{5}+8.75\;{\text{X}}_{4}{\text{X}}_{5}+7.81\;{\text{X}}_{11}^{2}-16.00\\ {\text{X}}_{22}^{2}-13.11\;{\text{X}}_{33}^{2}-5.67\;{\text{X}}_{44}^{2}-5.06\;{\text{X}}_{55}^{2} \end{array}$$

**Table 1:** ANOVA for Response Surface Quadratic model.

Source	SS	df	MS	F Value	p-value Prob > F
Model	1298492	20	64924.6	6.69169	< 0.0001*
Residual	281366	29	9702.27		
Lack of Fit	251724	22	11442	2.70205	0.0900
Pure Error	29641.9	7	4234.56		
Cor Total	1579857	49			

df—degrees of freedom, SS—sum of squares, MS—mean square,

* Significant p values, p ≤ 0.05; R^2^ = 0.8219; predicted R^2^ = 0.3551; Adjusted R^2^ = 0.6991

**Fig 8:**
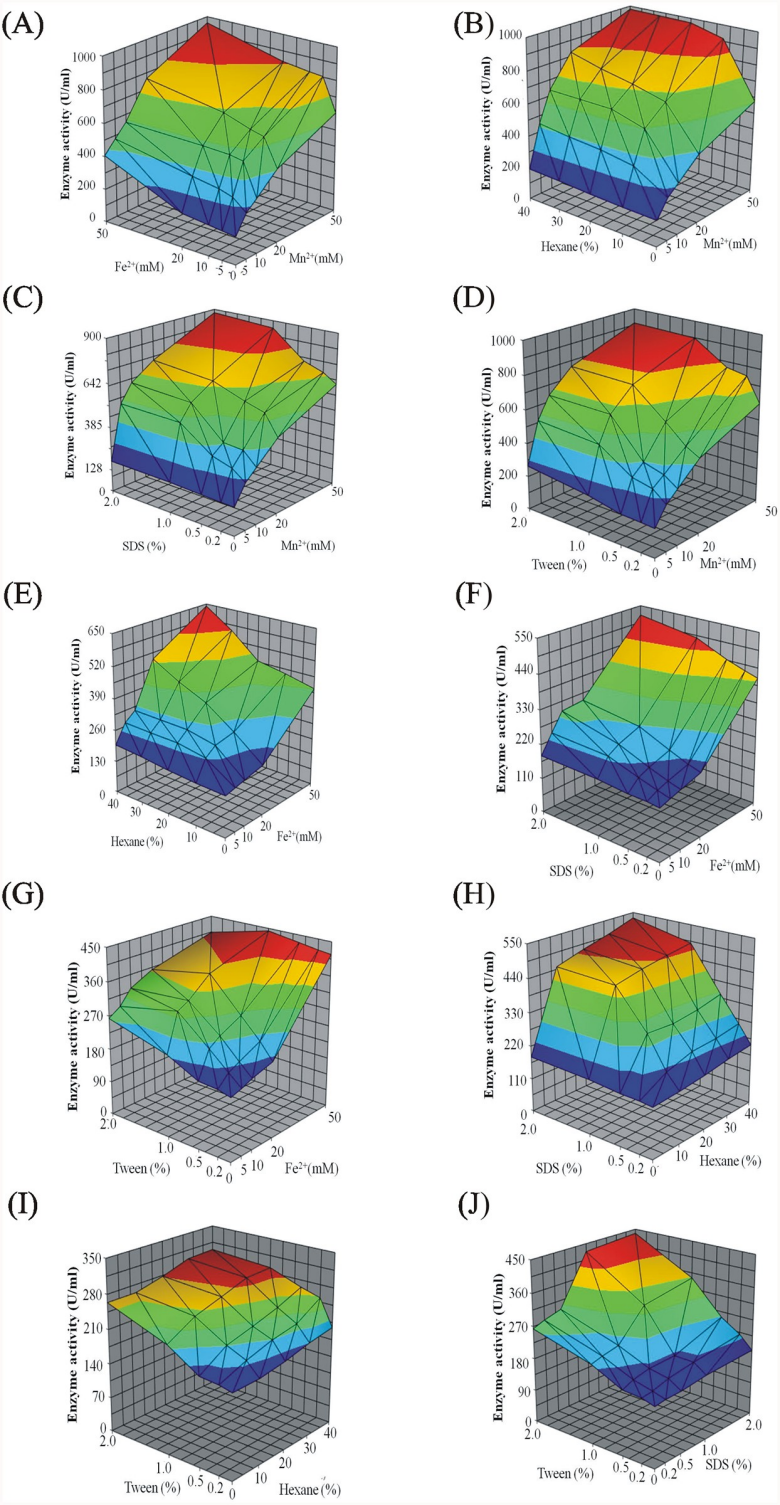
Response surface plots of activity of cold peptidase; varying factors were (A) Mn2+ and Fe2+, (B) Mn2+ and Hexane, (C) Mn2+ and SDS, (D) Mn2+ and Tween-80, (E) Fe2+ and Hexane, (F) Fe2+ and SDS, (G) Fe2+ and Tween-80, (H) Hexane and SDS, (I) Hexane and Tween-80, (J) SDS and Tween-80. Other variables were held at zero level and the residual activity of control was taken as 100%.

The results indicated that there was a significant effect of Mn^2+^, Fe^2+^, hexane, Tween-80 and SDS on the peptidase activity. The maximum peptidase activity predicted by the model was estimated to be 1232.8 U/mL. An experimental value of 1341.81 U/mL (peptidase activity) was obtained, which is in highly consistent with that of the predicted value and validates the model design. The three-dimensional surface plots represented the combined effect of two independent variables on peptidase activity, while the other three variables were maintained at zero level. There was a significant effect of interaction between the two variables on the activity of the enzyme (Fig 8).

## Discussion

The extracellular cold-active peptidase-producing bacterium isolated from a cold spring in the Himalayas belongs to *Bacillus sp*. A large number of peptidases available in the market have been isolated from different *Bacillus sp*. [13–18, 27]. Cold-active peptidases have been obtained from different psychrophilic and psychrotrophic bacteria [28–32]. In a previous study, using time course experiments, the maximum peptidase production by *B*. *subtilis* was reported at 48 hours [33]. The optimal pH value is above 7.0 for the proteases produced by various *Bacillus sp*. [34]. The maximal activity of the peptidases isolated from *Halobacterium sp*. and *Aeropyrum pernix* was at pH 8.0, and the peptidase produced by *Pseudoalteromonas sp*. had optimal enzyme activity at pH 8.5 [35–37]. In this study, the enzyme activity was greatly enhanced in the presence of Mn^2+^ and Fe^2+^. However, Ca^2+^, Cu^2+^ and Mg^2+^ decreased the enzyme activity of the peptidase produced by *Bacillus sp*. S1DI 10. Previous studies have reported a similar enhancement of peptidase activity in the presence of Mn^2+^ [38,39]. The activity of the peptidase isolated from *Halobacterium sp*. was reported to decline in the presence of Ca^2+^, Cu^2+^ and Mg^2+^ [35]. The activity of the serine peptidase isolated from *Thermoanaerobacter yonseiensis* was also inhibited in the presence of the Cu^2+^ metal ion [40]. The presence of organic solvent in the enzyme preparation is favored, since these can be used with water insoluble substrates and lower the risk of microbial contamination [41]. In our study, the peptidase activity of the enzyme increased up to 130% in the presence of hexane, while all the other organic solvents decreased the enzyme activity. It was observed that the enzyme was relatively stable in the presence of SDS, and the peptidase activity was enhanced up to 168% in Tween 80. In a previous study, cold-active peptidase isolated from *B*. *subtilis* showed similar characteristics in the presence of SDS and Tween 80 at low temperature [42]. In addition, the activity of peptidase was inhibited by Triton X-100 in this study. The inhibition of peptidase activity by Triton X-100 has also been reported previously [43,44]. In this study, the peptidase was inhibited in the presence of PMSF and leupeptin, suggesting that it is a serine peptidase. The peptidase activity was reversibly inhibited by EDTA, and the activity was reversed after the addition of Mn^2+^ ions. This property of the reversal of enzyme inhibition by metal ions implies that this peptidase is a metallopeptidase. Our studies also revealed that the enzymatic activity of *Bacillus sp*. S1DI 10 increased significantly in the presence of various factors, such as Mn^2+^, Fe^2+^, hexane, SDS and Tween-80, in a concentration-dependent manner. Three-dimensional response surfaces plots in this study show an increased enzyme activity with an increase in the concentration of two factors at a time. It was further observed that there was an initial increase in the activity of the enzyme upon an increase in the concentration of both Tween-80 and Fe2+, but at higher concentrations, a drop in enzyme activity was observed. CCD and RSM are powerful techniques used to test multiple process variables, since they require fewer experimental trials when compared with the study of one variable at a time. The CCD model and RSM were found to be very useful in determining the correlation between variables and the evaluation of their ideal concentrations. In other studies, RSM proved to be an important tool to identify increased enzyme production by *Bacillus sp*. strains. [45–48]. Using these techniques in our study, the model obtained was found to be very effective at optimizing the factors affecting the enzyme activity as indicated from the R^2^ value of 0.8219.

## Conclusion

The cold-active protease purified from *Bacillus sp*. S1DI 10 was detergent-stable with optimal enzymatic activity at 10°C and pH 8. The peptidase activity increased significantly following the addition of Mn^2+^ and Fe^2+^ metal ions and in the presence of the organic solvent hexane. The cold-active peptidases have numerous applications in food, leather, detergent and the pharmaceutical industry, as well as in the field of bioremediation. In household processes, the treatment of natural fabrics made of wool and silk with detergents active at low temperature can ensure the longevity of the product, while protecting their color and texture. In addition, the catalytic activity of the cold-active peptidase can be rapidly inactivated by mild heat treatment. This feature reduces the cost of energy inputs at the industrial scale and allows the cold active peptidases to be used as a tool for economic benefits.

## Supporting information

S1 Fig Zone of clearance on Skim mik agar indicating peptidase production by isolates. (TIF)

S2 Fig 16s rDNA amplified product of *Bacillus sp*. S1DI 10. (Lane 1–1 Kb DNA Ladder, Lane 2- 16s rDNA product of *Bacillus sp*. S1DI 10).
(TIF)

S3 Fig Peptidase production by *Bacillus sp*. S1DI 10 at different time intervals. (TIF)

S4 Fig Elution profile of cold peptidase (filled squares) and cold peptidase activity (filled circles) produced by *Bacillus sp*. S1DI 10. (TIF)

S5 Fig SDS PAGE analysis of purified peptidase from *Bacillus sp*. S1DI 10. (Lane 1 –Benchmark Pre-stained Protein Ladder (Invitrogen), Lane 2-Crude peptidase, Lane 3-Dialyzed peptidase, Lane 4-Purified concentrated peptidase).
(TIF)

S6 Fig Native PAGE analysis of purified peptidase from *Bacillus sp*. S1DI 10. (Lane 1 –Benchmark Pre-stained Protein Ladder (Invitrogen), 2 –Negative Control, 3 –Crude peptidase, 4 –Dialyzed peptidase, 5 –Purified peptidase).
(TIF)

S7 Fig **S7 (A) Fig**. PCR amplified cold peptidase gene—Lane 1 = 1 kb DNA Ladder, Lane 2 = Peptidase gene. **S7 (B) Fig.** Digested clone of cold peptidase gene—Lane 1 = 1 kb DNA Ladder, Lane 2 = double digested plasmid clone.
(TIF)

S8 Fig Phylogenetic tree of cold peptidase with the closely related peptidases. (TIF)

S9 Fig Lineweaver-burk plot of cold peptidase. (TIF)

S1 Table Biochemical characterization of the isolate S1DI 10. (PDF)

S2 Table Range and levels of independent variables with actual values. (PDF)

S3 Table Purification profile of cold peptidase from *Bacillus sp*. S1DI 10. (PDF)

S4 Table Experimental design, observed and predicted values of peptidase activity via CCD. (PDF)

S5 Table Statistical analysis of regression coefficients. (PDF)
