# Supplementary figures and images for: Characterization of a cold-active, detergent-stable metallopeptidase purified from Bacillus sp. S1DI 10 using Response Surface Methodology
Source: PLoS One. 2019 May 23;14(5):e0216990. doi: 10.1371/journal.pone.0216990 (PMC6532869; doi:10.1371/journal.pone.0216990)

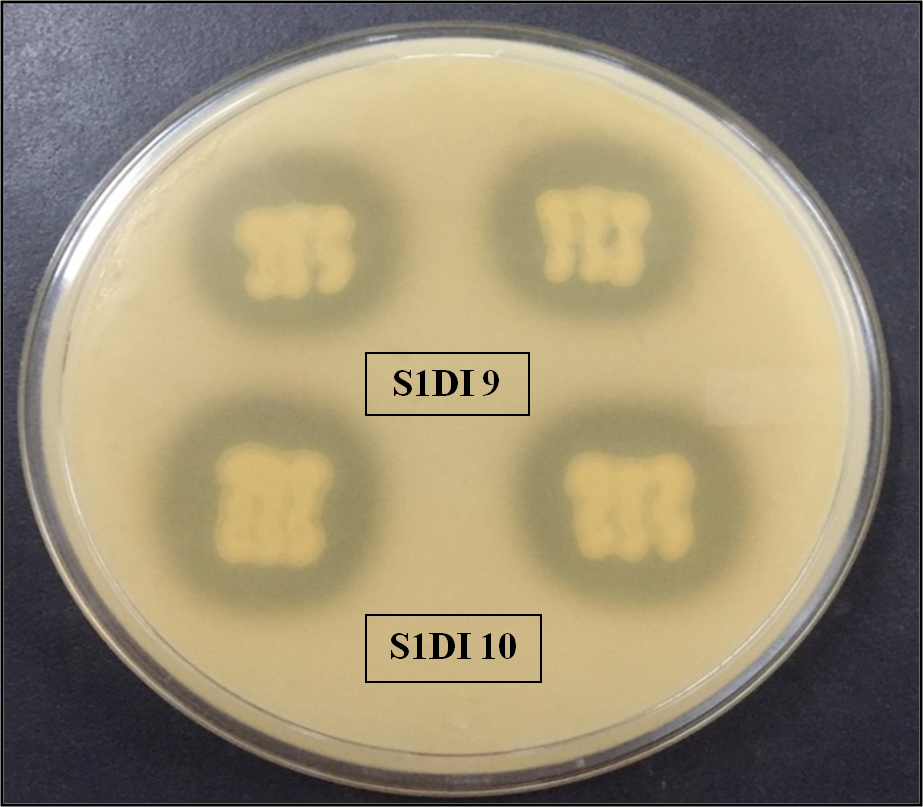

Supplement: S1 Fig — (TIF) [file pone.0216990.s001.tif]

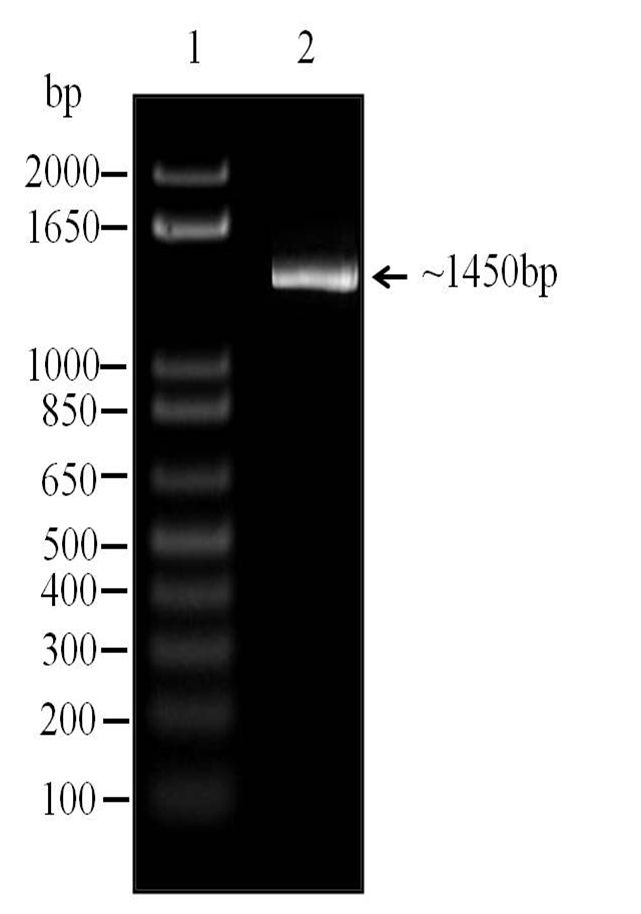

Supplement: S2 Fig — (Lane 1–1 Kb DNA Ladder, Lane 2- 16s rDNA product of Bacillus sp. S1DI 10). (TIF) [file pone.0216990.s002.tif]

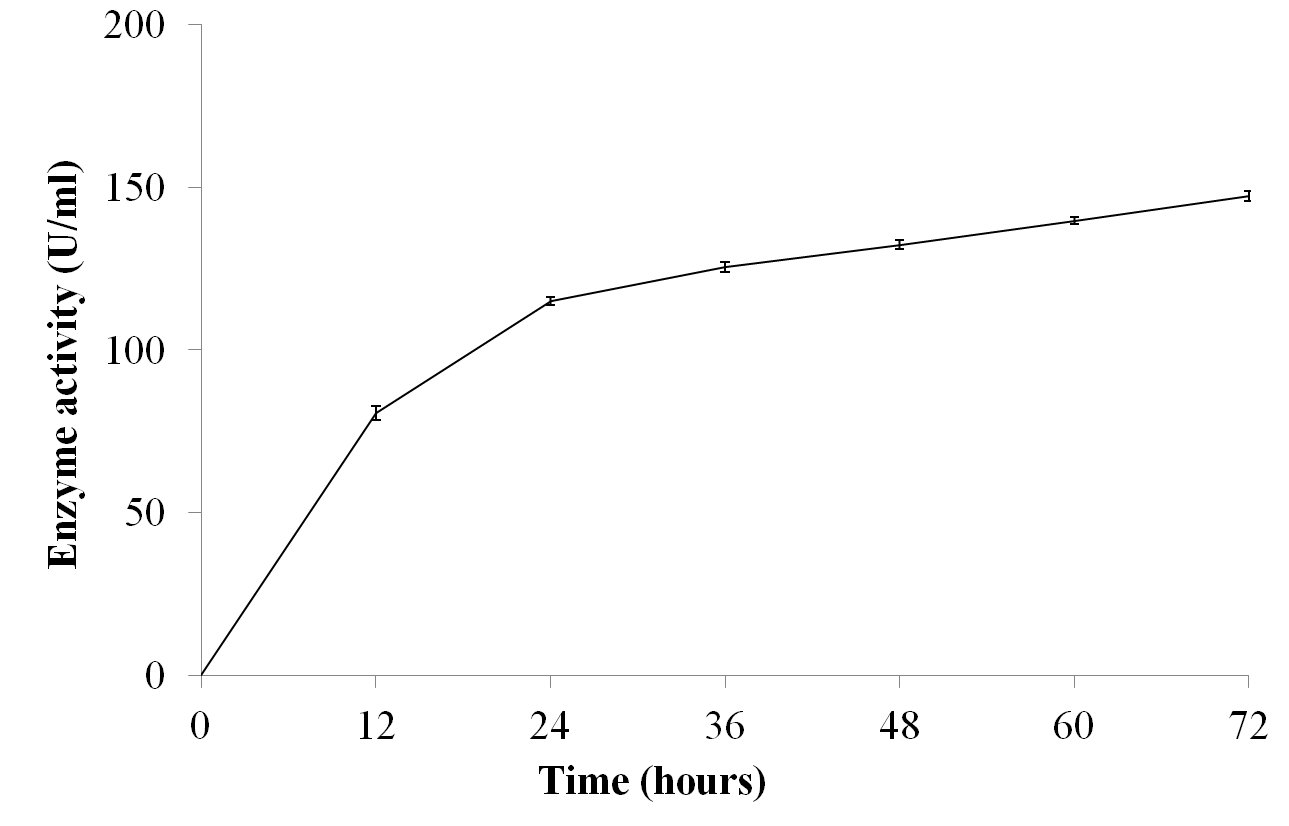

Supplement: S3 Fig — (TIF) [file pone.0216990.s003.tif]

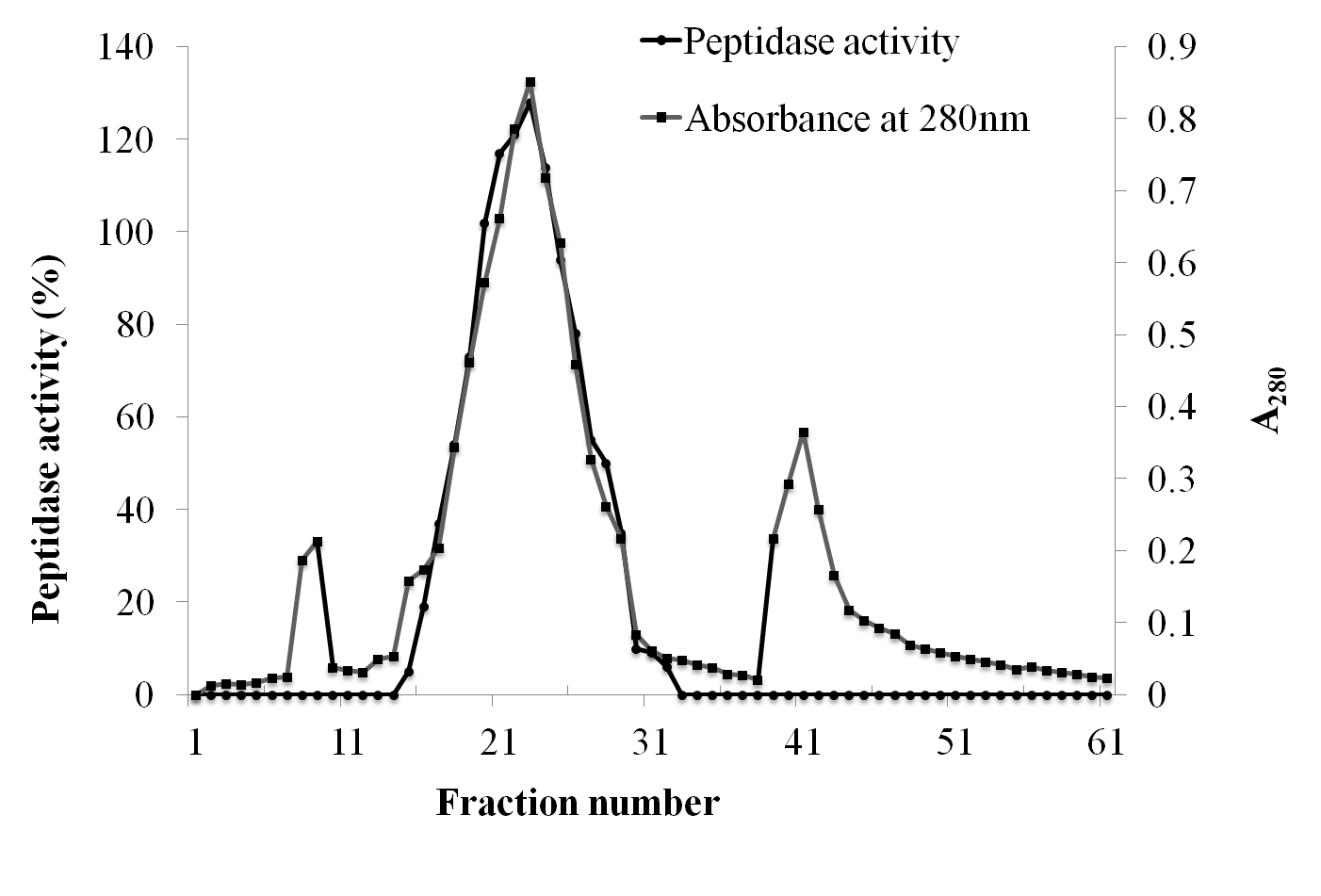

Supplement: S4 Fig — (TIF) [file pone.0216990.s004.tif]

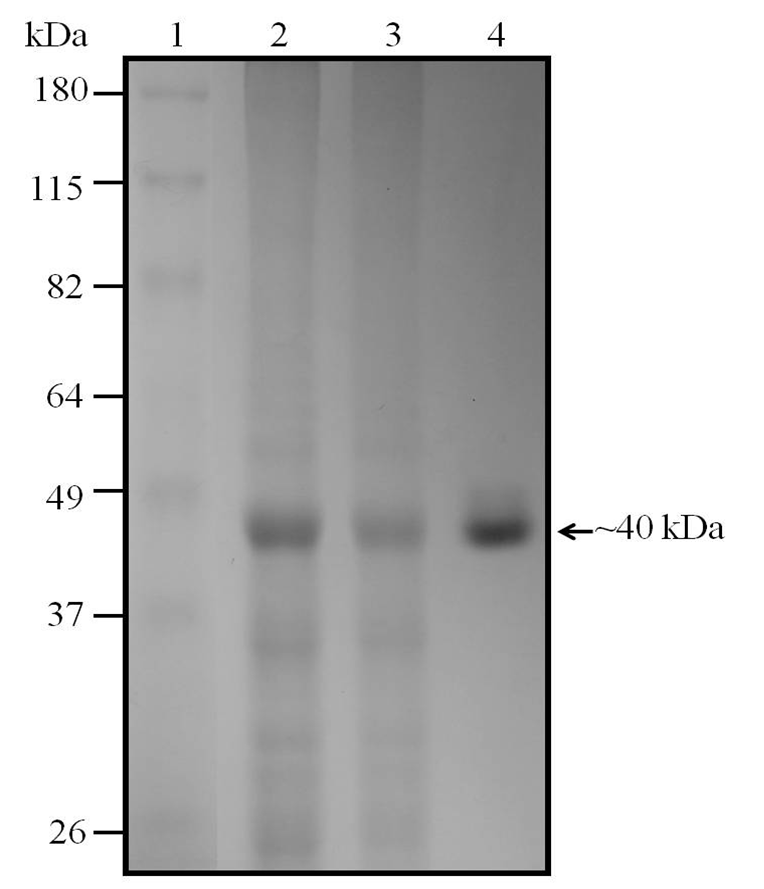

Supplement: S5 Fig — (Lane 1 –Benchmark Pre-stained Protein Ladder (Invitrogen), Lane 2-Crude peptidase, Lane 3-Dialyzed peptidase, Lane 4-Purified concentrated peptidase). (TIF) [file pone.0216990.s005.tif]

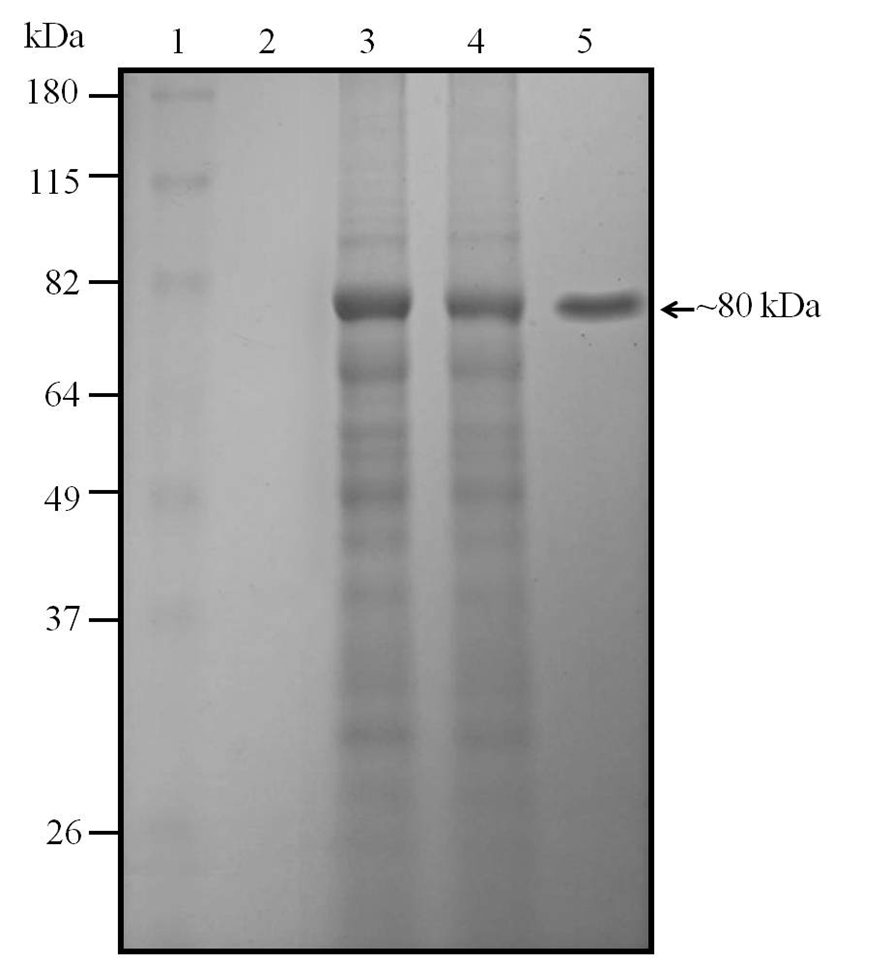

Supplement: S6 Fig — (Lane 1 –Benchmark Pre-stained Protein Ladder (Invitrogen), 2 –Negative Control, 3 –Crude peptidase, 4 –Dialyzed peptidase, 5 –Purified peptidase). (TIF) [file pone.0216990.s006.tif]

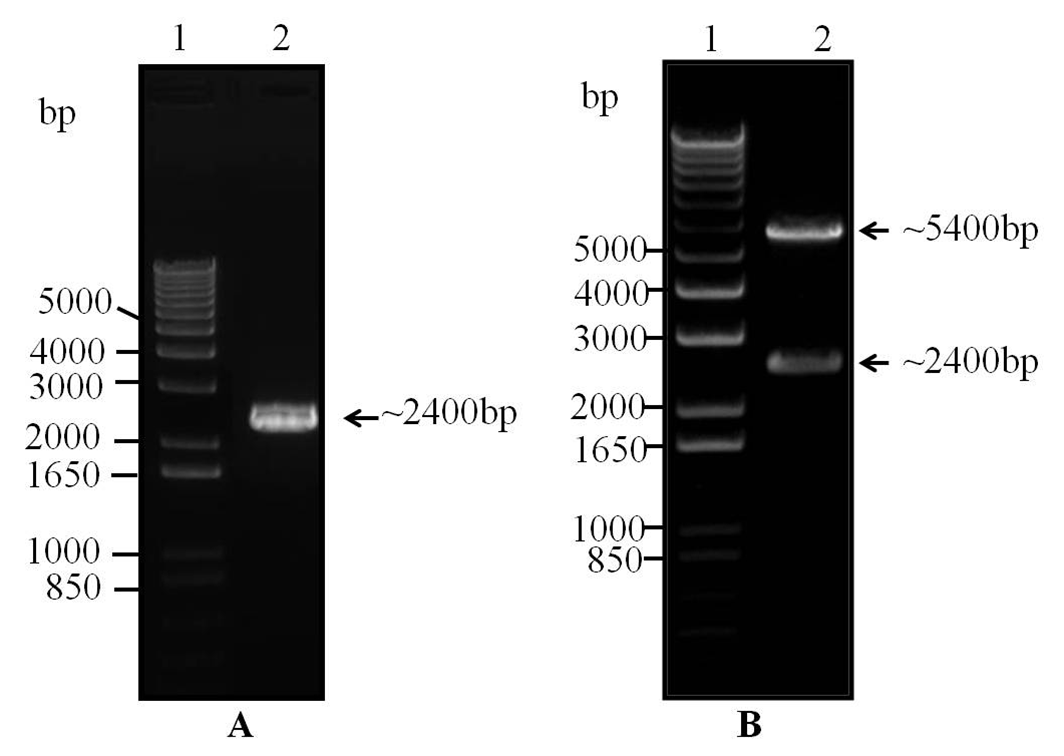

Supplement: S7 Fig — S7 (A) Fig. PCR amplified cold peptidase gene—Lane 1 = 1 kb DNA Ladder, Lane 2 = Peptidase gene. S7 (B) Fig. Digested clone of cold peptidase gene—Lane 1 = 1 kb DNA Ladder, Lane 2 = double digested plasmid clone. (TIF) [file pone.0216990.s007.tif]

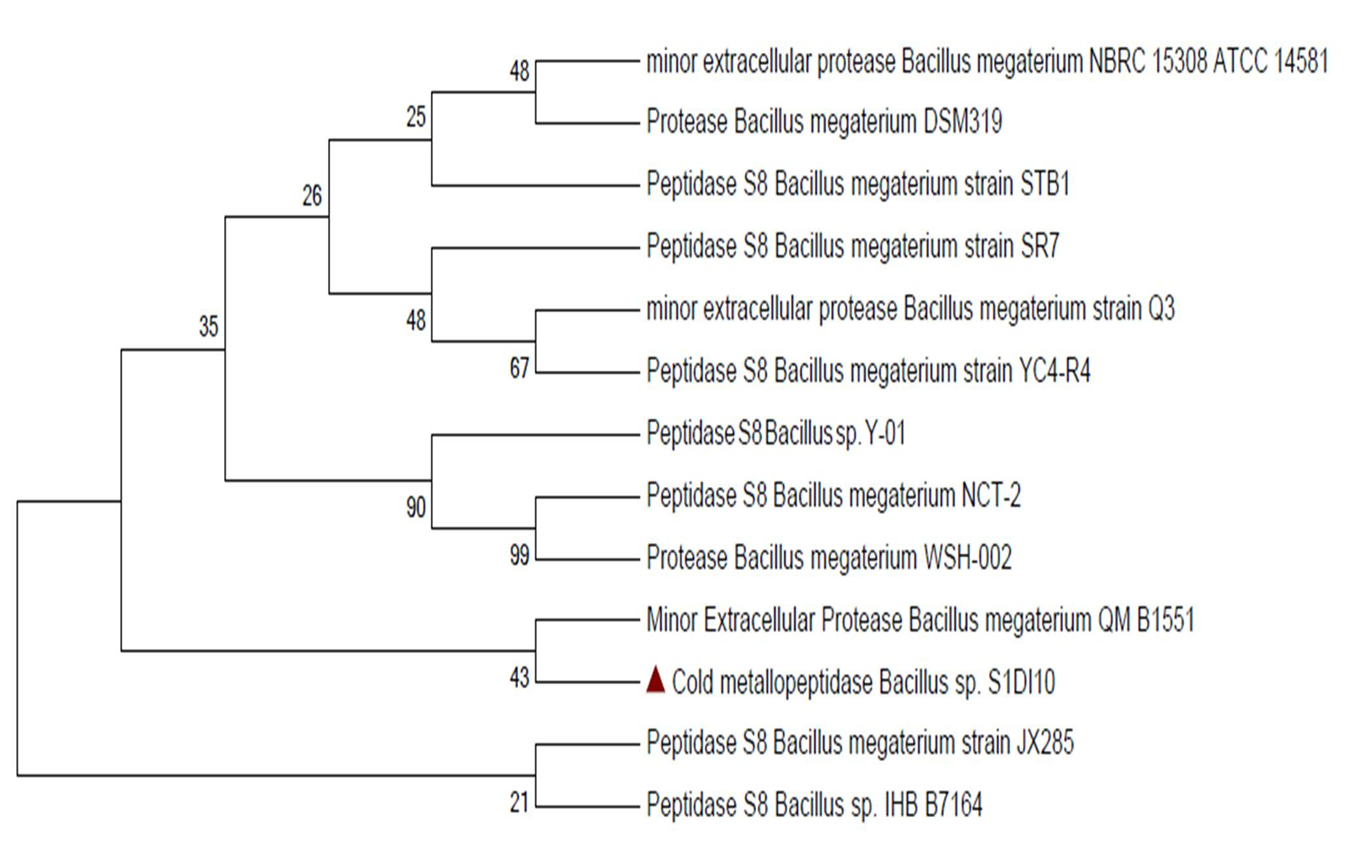

Supplement: S8 Fig — (TIF) [file pone.0216990.s008.tif]

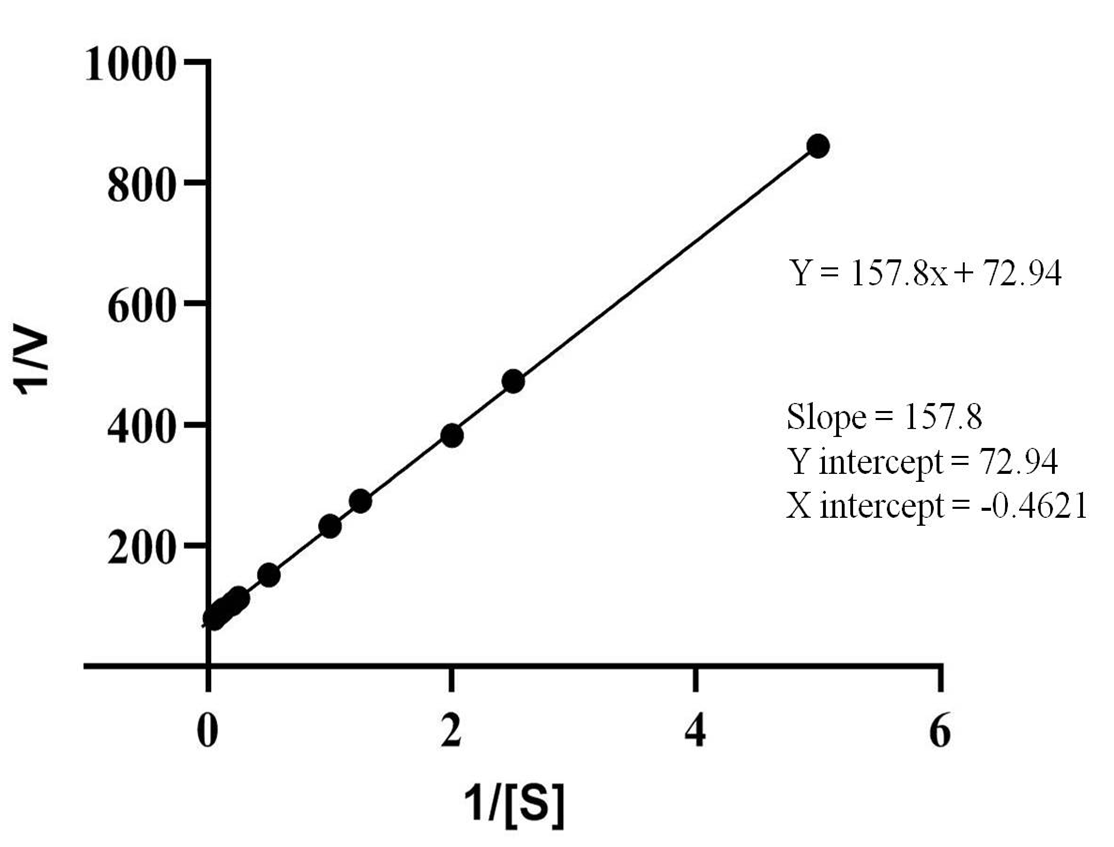

Supplement: S9 Fig — (TIF) [file pone.0216990.s009.tif]
