# Supplementary material for: Characterization of a cold-active, detergent-stable metallopeptidase purified from Bacillus sp. S1DI 10 using Response Surface Methodology
Source: PLoS One. 2019 May 23;14(5):e0216990. doi: 10.1371/journal.pone.0216990 (PMC6532869; doi:10.1371/journal.pone.0216990)
Supplement: S1 Table — (PDF) [file pone.0216990.s010.pdf]

S1 Table. Biochemical characterization of the isolate S1DI 10.

S. No.	Biochemical Test	Result
1.	Gram's	+
2.	Indole	+
3.	Methyl Red (MR)	-
4.	Voges Proskauer (VP)	-
5.	Oxidase	+
6.	Catalase	-
7.	Simmon's citrate	-
8.	Urease	+
9.	Motility	+
10.	Oxidation	+
11.	Fermentation	-
12.	Triple Sugar Iron (TSI)	+
13.	H_2S production	-
14.	Glucose	+
15.	Sucrose	+
16.	Lactose	+
17.	Mannitol	+
