# Supplementary material for: Characterization of a cold-active, detergent-stable metallopeptidase purified from Bacillus sp. S1DI 10 using Response Surface Methodology
Source: PLoS One. 2019 May 23;14(5):e0216990. doi: 10.1371/journal.pone.0216990 (PMC6532869; doi:10.1371/journal.pone.0216990)
Supplement: S2 Table — (PDF) [file pone.0216990.s011.pdf]

S2 Table. Range and levels of Independent variables with actual values

		**Range and Levels**				
**S. No.**	**Independent Variables**	**-2**	**-1**	**0**	**+1**	**+2**
1	Mn^{2+} (mM)	0	5	10	20	50
2	Fe^{2+}(mM)	0	5	10	20	50
3	Hexane (%)	0	10	20	30	40
4	SDS (%)	0	0.2	0.5	1	2
5	Tween 80 (%)	0	0.2	0.5	1	2
