# Supplementary material for: Characterization of a cold-active, detergent-stable metallopeptidase purified from Bacillus sp. S1DI 10 using Response Surface Methodology
Source: PLoS One. 2019 May 23;14(5):e0216990. doi: 10.1371/journal.pone.0216990 (PMC6532869; doi:10.1371/journal.pone.0216990)
Supplement: S3 Table — (PDF) [file pone.0216990.s012.pdf]

S3 Table. Purification profile of Cold peptidase from *Bacillus sp.* S1DI 10

Purification steps	Total activity (U)	Total protein (mg)	Specific activity (U/mg)	Purification fold	Yield (%)
**Culture supernatant**	1841	48.4	38.03	1	100
**Partially purified**	1273	14.3	89.02	2.34	69.14
**Sephadex G100**	316	2.23	141.7	3.72	17.16
