# Supplementary material for: Characterization of a cold-active, detergent-stable metallopeptidase purified from Bacillus sp. S1DI 10 using Response Surface Methodology
Source: PLoS One. 2019 May 23;14(5):e0216990. doi: 10.1371/journal.pone.0216990 (PMC6532869; doi:10.1371/journal.pone.0216990)
Supplement: S4 Table — (PDF) [file pone.0216990.s013.pdf]

S4 Table. Experimental design, Observed and Predicted values of Peptidase activity via CCD

**Independent variables, Codes and Values**						**Peptidase activity (Units/ml)**	
Run no.	Mn^{2+} (X_1)	Fe^{2+} (X_2)	Hexane (X_3)	SDS (X_4)	Tween-80 (X_5)	Obtained	Predicted
1	-1	-1	-1	-1	-1	360.57	476.22
2	1	-1	-1	-1	-1	669.63	604.82
3	-1	1	-1	-1	-1	540.85	522.09
4	1	1	-1	-1	-1	721.14	694.22
5	-1	-1	1	-1	-1	571.76	543.03
6	1	-1	1	-1	-1	705.68	705.36
7	-1	1	1	-1	-1	618.12	596.22
8	1	1	1	-1	-1	759.74	802.09
9	-1	-1	-1	1	-1	587.21	537.82
10	1	-1	-1	1	-1	733.99	744.59
11	-1	1	-1	1	-1	630.97	599.4
12	1	1	-1	1	-1	798.4	849.7
13	-1	-1	1	1	-1	664.47	639.14
14	1	-1	1	1	-1	826.71	879.64
15	-1	1	1	1	-1	682.48	708.05

16	1	1	1	1	-1	888.52	992.08
17	-1	-1	-1	-1	1	594.91	546.92
18	1	-1	-1	-1	1	728.84	729.36
19	-1	1	-1	-1	1	654.17	645.98
20	1	1	-1	-1	1	814.36	871.95
21	-1	-1	1	-1	1	674.78	641.69
22	1	-1	1	-1	1	767.49	857.86
23	-1	1	1	-1	1	718.53	748.07
24	1	1	1	-1	1	916.87	1007.78
25	-1	-1	-1	1	1	630.97	643.56
26	1	-1	-1	1	1	860.21	904.17
27	-1	1	-1	1	1	754.59	758.32
28	1	1	-1	1	1	955.48	1062.46
29	-1	-1	1	1	1	715.98	772.84
30	1	-1	1	1	1	996.69	1067.18
31	-1	1	1	1	1	759.74	894.92
32	1	1	1	1	1	1341.81	1232.8
33	-2	0	0	0	0	566.61	588.84
34	2	0	0	0	0	1236.24	1055.31
35	0	-2	0	0	0	643.87	621.03
36	0	2	0	0	0	968.38	832.52

37	0	0	-2	0	0	618.12	619.78
38	0	0	2	0	0	1017.29	856.92
39	0	0	0	-2	0	633.57	624.77
40	0	0	0	2	0	1061.31	911.4
41	0	0	0	0	-2	602.66	614.85
42	0	0	0	0	2	1097.16	926.27
43	0	0	0	0	0	834.46	790.81
44	0	0	0	0	0	746.89	790.81
45	0	0	0	0	0	695.38	790.81
46	0	0	0	0	0	664.47	790.81
47	0	0	0	0	0	878.22	790.81
48	0	0	0	0	0	798.4	790.81
49	0	0	0	0	0	687.63	790.81
50	0	0	0	0	0	762.34	790.81
