# Supplementary material for: Characterization of a cold-active, detergent-stable metallopeptidase purified from Bacillus sp. S1DI 10 using Response Surface Methodology
Source: PLoS One. 2019 May 23;14(5):e0216990. doi: 10.1371/journal.pone.0216990 (PMC6532869; doi:10.1371/journal.pone.0216990)
Supplement: S5 Table — (PDF) [file pone.0216990.s014.pdf]

S5 Table. Statistical analysis of Regression coefficients

Factor	Coefficient Estimate	Standard Error	F Value	p-value Prob > F
Intercept	790.811	34.1214298	56.0684	< 0.0001
A-Mn^{2+}	116.618	15.57423066	11.5251	0.0020
B-Fe^{2+}	52.8725	15.57423066	14.4905	0.0007
C-Hexane	59.2855	15.57423066	21.1689	< 0.0001
D-SDS	71.6565	15.57423066	24.9893	< 0.0001
E-Tween 80	77.8545	15.57423066	0.39069	0.5368
AB	10.8838	17.41251923	0.23453	0.6318
AC	8.4325	17.41251923	1.25953	0.2709
AD	19.5419	17.41251923	0.59748	0.4458
AE	13.4594	17.41251923	0.01107	0.9169
BC	1.83188	17.41251923	0.05084	0.8232
BD	3.92625	17.41251923	0.58309	0.4513
BE	13.2963	17.41251923	0.2455	0.6240
CD	8.6275	17.41251923	0.16109	0.6911
CE	6.98875	17.41251923	0.25299	0.6188
DE	8.75813	17.41251923	0.20152	0.6568
A^2	7.81663	17.41251923	0.84522	0.3655
B^2	-16.008	17.41251923	0.56716	0.4575

C^2	-13.113	17.41251923	0.10639	0.7466
D^2	-5.6796	17.41251923	0.08452	0.7733
E^2	-5.0621	17.41251923	0.02066	0.8867

* Significant p values, $p \leq 0.05$
